# Delineating impulsivity-based pathways to suicide deaths: A cluster analysis

**DOI:** 10.1192/j.eurpsy.2025.10070

**Published:** 2025-08-11

**Authors:** Sergio Sanz-Gomez, Adrián Alacreu-Crespo, Elia Gourgechon-Buot, Maria Isabel Perea-Gonzalez, Jorge Luis Ordoñez-Carrasco, Philippe Courtet, Lucas Giner

**Affiliations:** 1 Department of Psychiatry, Universidad de Sevilla, Sevilla, Spain; 2 Department of Psychology and Sociology, Universidad de Zaragoza, Zaragoza, Spain; 3 IGF, Univ. Montpellier, CNRS, INSERM, Montpellier, France; 4 Department of Emergency Psychiatry and Acute Care, Lapeyronie Hospital, CHU Montpellier, Montpellier, France

**Keywords:** aggression, impulsivity, psychological autopsy, suicide, suicide deaths

## Abstract

**Background:**

The significant heterogeneity among individuals who die by suicide complicates prevention, suggesting that a “one-size-fits-all” approach is insufficient. It is crucial to identify distinct subgroups for targeted strategies. This study aims to characterize suicide profiles based on trait impulsivity and related factors.

**Methods:**

Data from the FRieNDS project (*Factores de Riesgo en Defunciones por Suicidio –* Risk Factors in Suicide Deaths), a psychological autopsy study of 408 suicide deaths, were used. After determining the optimal number of clusters via stability analysis through agglomerative nesting, a final cluster analysis was performed on 391 valid suicide deaths (defined as cases with no missing data on the variables used for clustering) using *k*-means on a lower-dimensional representation of the data encoded by an autoencoder. Key clustering variables included sex, impulsivity (Barratt Impulsivity Scale-11), aggression, intent to die, previous history of suicide attempts, history of substance abuse, psychotic and affective disorders, and the presence of a depressive episode at the time of death.

**Results:**

We identified three clusters: (1) Impulsive-aggressive (29.8%), characterized by high rates of Cluster B disorders, substance abuse, more stressful events, and low lethal intent; (2) depressive prior attempters (24.5%), which comprised mostly women and showed greater behavioural changes before death; and (3) non-impulsive/aggressive (45.7%), a group with no clear psychopathological profile, less healthcare contact, and minimal communicated intent to die, despite having few prior attempts.

**Conclusions:**

Our study identified three suicide clusters with varying impulsivity levels, highlighting the need for tailored interventions and community-level research for better suicide prevention strategies.

## Introduction

Global estimates indicate that suicide accounts for between 692,000 and 800,000 deaths per year [1]. Still, suicide remains a major public health challenge due to its multifactorial nature, involving genetic, neurophysiological, psychological, social, and cultural factors [2]. These complexities hinder prevention efforts. While psychiatric disorders are strongly associated with suicide [3], a significant proportion of cases occur without identified pathology [4]. Prior attempts are the strongest predictor of future suicide [5, 6], yet most suicides occur on the first attempt, limiting their predictive value [7]. Sex differences introduce further complexities: for instance, males who die by suicide are less likely than their female counterparts to have a diagnosed mental disorder and more likely to have experienced life stressors before death [8–10].

Impulsivity is closely linked to suicidal behaviour [11, 12]. Psychological autopsy studies consistently report higher trait impulsivity in suicide decedents compared to both living community controls [13–21] and individuals with similar clinical profiles [22, 23] or sudden deaths due to disease or accidents [24]. Among adults who die by suicide, high levels of impulsivity are present in both men and women, even if the prevalence is generally lower in females [18, 25]. However, impulsivity is higher in attempters than in those who die by suicide [26].

Many suicides occur in individuals with low impulsivity, who display unique characteristics. In terms of psychopathology, this group often presents with less overall diagnosed psychopathology, particularly a lower likelihood of Cluster B personality disorders and substance or alcohol use disorders [27, 28]. However, some research has linked this profile to a higher likelihood of depression [29]. Paradoxically, this lower externalizing profile is associated with a higher intent to die [28, 29], which may involve methods of high lethality [30]. Therefore, understanding this low-impulsivity profile is essential, as these individuals may be overlooked by traditional risk assessment tools that are heavily focused on overt crisis behaviours [31].

These findings underscore a key challenge in suicide prevention. Despite its multifactorial nature, distinct risk and protective factors likely apply to different subgroups. Evidence for suicidal subtypes emerges from two observations: (1) inconsistent relationships between risk factors and suicidal behaviour, and (2) associations between clinical profiles and neurobiological patterns [32]. A systematic review and meta-analysis suggest two primary subtypes of suicide decedents: impulsive and non-impulsive [33]. However, this dichotomy is further nuanced by the interplay of other critical factors. For instance, the presence of severe depression can significantly influence suicidal planning and intent regardless of impulsivity levels [30, 34], while acute life stressors may further impact risk for fatal suicide behaviour in this population [35]. Recognizing these distinctions is crucial for effective prevention and intervention.

### Rationale and objectives

This study aims to characterize suicide decedents based on trait impulsivity using psychological autopsy methods. We hypothesize two groups: (i) a highly impulsive group, expected to be younger, with higher Cluster B personality and substance use disorders, and lower intent to die; and (ii) a low impulsive group, anticipated to be older, with more depressive and schizophrenia spectrum disorders, and higher intent to die.

Additionally, these groups are expected to differ in suicide pathways. The highly impulsive group is likely to face more recent negative life events and financial or work problems, while the low impulsive group may have more health issues and reduced social support. Gender differences will also be examined, as low impulsivity is a suicide risk factor in depressed women [36].

Beyond these hypotheses, we will explore potential intermediate profiles, refining impulsivity-based suicide risk models. The variables align with prior systematic review findings [33] and the study database [37].

## Methods

### Sample description

This study utilized data from the FRienDS research project (*Factores de Riesgo en Defunciones por Suicidio –* Risk Factors in Suicide Deaths) [26]. Psychological autopsies were conducted through structured interviews with relatives or close contacts of the deceased. Ethical approval was granted by the Ethics and Health Research Committee of the Virgen Macarena Hospital Area in Seville, registry number 982, adhering to the Declaration of Helsinki. The sample comprised 408 suicide decedents from Seville, Spain (2006–2018).

### Procedure

Forensic doctors informed relatives about the study at the Pathology Service, explaining objectives and methodology before seeking consent. Participants received a consent form with study details and a contact number for further inquiries or withdrawal. Families were approached at least 9 months postmortem. This waiting period was implemented both to reduce the potential for recall bias in the data and, crucially, as an ethical consideration to protect the families during their most difficult time of mourning. Consequently, most interviews were conducted 12–18 months after death.

### Measures

Psychological autopsies were conducted by trained psychiatrists or health psychologists, all of whom had completed at least five autopsies under senior supervision. Interviews lasted 2–4 h, following a structured protocol [38].

Data collection included sociodemographic variables, suicide history, negative life events (past year), healthcare use (final 3 months), behavioural changes (last 2 weeks), and attitudes towards death. Psychiatric diagnoses were obtained using the Diagnostic and Statistical Manual of Mental Disorders, Fourth Edition, Text Revision (DSM-IV-TR) Structured Clinical Interview for DSM Disorders (SCID I and II) [39, 40]. The SCID-I was used for Axis I disorders, whereas the SCID-II was used for personality disorders. Both interviews were administered by trained clinicians who established diagnoses based on fulfillment of DSM-IV-TR diagnostic criteria.

- Barratt Impulsivity Scale-11: A 30-item Spanish version assessing trait impulsivity [41, 42]. Items are rated on a 4-point Likert scale (range: 30–120), covering cognitive, unplanned, and motor impulsivity. In the current sample, the scale demonstrated excellent internal consistency (Cronbach’s *α* = 0.889)
- Brown–Goodwin Life History of Aggression (BGLHA): Evaluates aggression across childhood, adolescence, and adulthood through 11 areas (e.g., school discipline, antisocial behaviour, self-injury), scored on a 4-point Likert scale (range: 0–44) [43, 44]. The internal consistency in our sample was also excellent (Cronbach’s *α* = 0.946).
- Beck Suicidal Intent Scale (SIS): An 18-item (range: 0–36), interviewer-rated tool assessing intent during self-harm, validated in Spanish (Cronbach’s *α* = 0.8) [45, 46].
- Paul Ramsey Life Experience Scale: Rates acute stressful events over 6 months on a 7-point Likert severity scale (range: 0–49), with excellent test–retest reliability (*r* = .95) [47].
- Holmes and Rahe Social Adjustment Scale: Measures chronic stress from 46 life events over a year, using the Spanish version [48, 49]. The scale is a checklist of life events, where each event is assigned a specific weighted score known as a Life Change Unit (LCU). The total score is the sum of the LCUs for all events experienced by the individual in the past year.
- Life Threatening Events Questionnaire (LTE-Q): Lists 12 major life events (e.g., job loss and illness), summing checked events (range: 0–12). The Spanish adaptation shows good reliability (*κ* = 0.61–0.83) [50, 51].

### Statistical analysis

All statistical analyses were conducted using R version 4.4.1 [52]. Cluster analysis was performed on 391 valid suicide cases, first encoding data into a lower-dimensional representation via autoencoders [53], then clustering with *k*-means. The number of autoencoder neurons and *k*-means clusters was optimized for cluster-wise stability, assessed through bootstrap resampling (1,000 samples) and Jaccard similarity [54]. A solution was considered highly stable if the mean Jaccard similarity exceeded 0.85; the selected three-cluster solution demonstrated excellent stability for the total population (mean Jaccard = 0.87) and for the sex-segregated analyses of males (0.94) and females (0.99).

Cluster variables included: sex, age, BIS, BGLHA, SIS, lifetime suicide attempts (none, one, two, or more), and psychiatric diagnoses, each treated as a binary (presence/absence) variable: depressive disorder, psychotic disorder, anxiety disorder, substance abuse disorder, and DSM-IV Cluster A personality disorder, DSM-IV Cluster B personality disorder, and DSM-IV Cluster C personality disorder. Key continuous variables, such as impulsivity and aggression scores, were included without prior categorization to preserve the full range of variance and allow the data-driven algorithm to identify natural groupings without imposing clinical thresholds.

Clusters derived from our analysis were named based on significant attributes, with sex-separated analyses conducted for men and women.

Cluster differences were assessed via a multinomial model, followed by pairwise logistic models. To account for multiple comparisons, *p*-values from all models were adjusted using the false discovery rate correction with the Benjamini–Hochberg procedure; these adjusted values are reported as *q*-values throughout the tables. Additional examined variables included the following:

- Sociodemographic: Marital status, children, education, employment, income, economic dependents, living alone, and social contact.
- Negative life events: Holmes–Rahe scale, Paul Ramsey scale, and LTE-Q.
- Healthcare contact (last 3 months): General practitioner, psychiatrist, emergency psychiatrist, and psychologist.
- Warning signs: Suicidal thoughts/intent, hospital referral, and family warning.
- Behavioural changes (last 2 weeks): Work engagement, task quality, work shame, social conflicts, anger, worry, and reduced social contact.
- Attitudes towards death/suicide: Preference for death over aging/being a burden, wish to die, and suicide reasons/method.

## Results

### Cluster analysis

**Figure 1.**
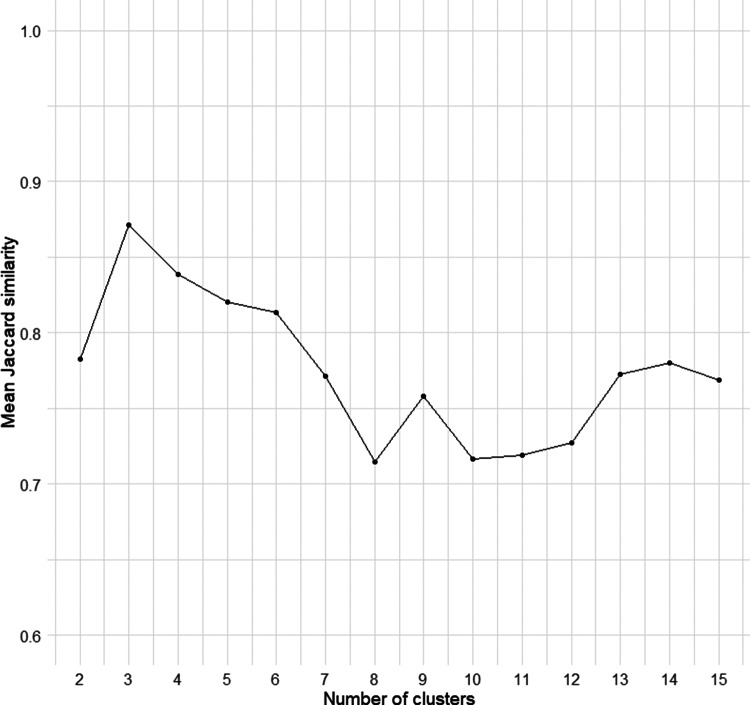
Determination of the optimal number of clusters using stability analysis. This plot displays the mean Jaccard similarity for cluster solutions ranging from *k* = 2 to *k* = 15, obtained through bootstrap resampling. The Jaccard similarity coefficient measures the stability and reproducibility of the clusters. The three-cluster solution was selected as optimal because it yielded the highest mean Jaccard similarity, indicating the most stable classification of the data.

**Figure 2.**
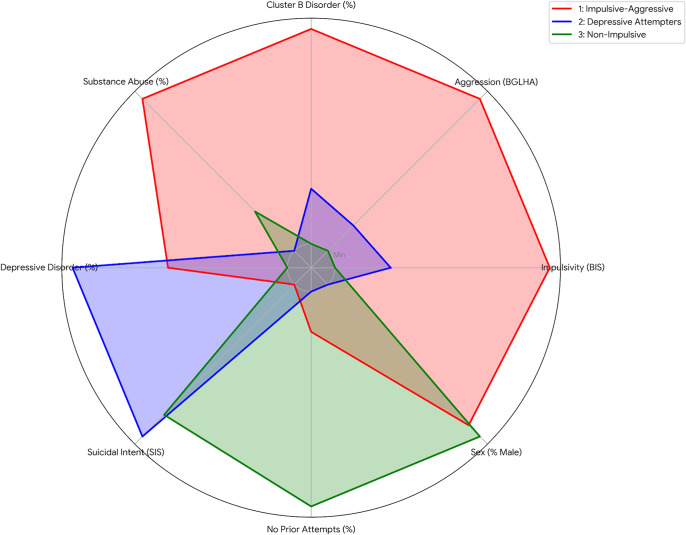
Radar plot of cluster profiles. *Note*: BGLHA, Brown–Goodwin Life History of Aggression; BIS, Barratt Impulsiveness Scale; SIS, Suicide Intent Scale. This plot provides a visual representation of the three cluster profiles across eight key variables. To allow for comparison across scales with different ranges, values for each variable have been normalized and then shifted so that the minimum value is plotted at a visible baseline (labelled “Min”) instead of the centre (*r* = 0). This method highlights the relative strengths and weaknesses of each profile while ensuring all cluster shapes are fully visible.

The optimal number of clusters was determined using mean Jaccard similarity (Figure 1). Cluster analysis identified three distinct profiles among suicide decedents. See Table 1 for comprehensive characteristics and Figure 2 for a visual representation of the cluster profiles.

- The first profile, termed “Impulsive-aggressive” (*n* = 108; 27.6% of the sample), was distinguished by the highest levels of impulsivity and aggression, an elevated prevalence of substance abuse, and lower suicidal intent compared to the other two clusters. This cluster was mostly male (*n* = 100; 93% of the cluster).
- The second profile, labelled “Depressive prior attempters” (*n* = 105; 26.6% of the sample), exhibited the highest prevalence of current depressive episodes and a notable proportion of individuals with a history of suicide attempts. This group presented lower impulsivity and aggression than Cluster 1, while their suicidal intent was higher than that of Cluster 1 and comparable to that of Cluster 3. This cluster had the lower proportion of men (*n* = 19; 18%). Finally, the third profile, named “Non-impulsive/aggressive cases with no prior attempts” (*n* = 178; 45.5% of the sample), was characterized by the lowest levels of impulsivity and aggression. Most of its members had no history of previous suicide attempts, markedly distinguishing it from the other two profiles. This cluster comprised almost entirely of men (*n* = 177; 99%).

**Table 1.** Characterization of the cluster according to defining variables

Characteristic	Distribution			Multinomial model			1 vs. 2	1 vs. 3	2 vs. 3
	Impulsive-aggressive,*N* = 108	Depressive prior attempters,*N* = 105	Non-impulsive-aggressive,*N* = 178	OR (CI)1 vs. 2	OR (CI)1 vs. 3	Global *q*-value^a^	*q*-value^b^	*q*-value^b^	*q*-value^b^
Sex						**<0.001**			
*Male*	100 (93%)	19 (18%)	177 (99%)	—	—				
*Female*	8 (7.4%)	86 (82%)	1 (0.6%)	56.6, (23.6,136)	0.07, (0.01,0.57)		**<0.001**	**0.039**	**<0.001**
Age	50 (17)	56 (17)	55 (22)	1.02, (1.00,1.03)	1.01, (1.00,1.03)	0.051	**0.037**	0.13	>0.9
BIS total	69 (25)	49 (25)	42 (22)	0.97, (0.96,0.98)	0.96, (0.94,0.97)	**<0.001**	**<0.001**	**<0.001**	**0.038**
BGHA score	26 (21)	11 (13)	8 (11)	0.95, (0.93,0.97)	0.93, (0.91,0.95)	**<0.001**	**<0.001**	**<0.001**	0.2
Number of SA						**<0.001**			
*Two or more*	38 (35%)	42 (40%)	8 (4.5%)	—	—				
*One*	26 (24%)	29 (28%)	28 (16%)	1.01, (0.51,2.01)	5.12, (2.02,13.0)		>0.9	**0.002**	**0.002**
*None*	44 (41%)	34 (32%)	142 (80%)	0.70, (0.37,1.31)	15.3, (6.66,35.3)		0.8	**<0.001**	**<0.001**
SIS score	21 (9)	28 (7)	27 (7)	1.10, (1.06,1.14)	1.09, (1.06,1.12)	**<0.001**	**<0.001**	**<0.001**	>0.9
Depressive disorder – current	69 (64%)	80 (76%)	88 (49%)	1.81, (1.00,3.28)	0.55, (0.34,0.90)	**<0.001**	0.2	0.053	**<0.001**
Any psychotic spectrum disorder	17 (16%)	34 (32%)	30 (17%)	2.56, (1.33,4.96)	1.09, (0.57,2.08)	**0.004**	**0.015**	>0.9	**0.009**
Substance abuse	78 (72%)	9 (8.6%)	44 (25%)	0.04, (0.02,0.08)	0.13, (0.07,0.22)	**<0.001**	**<0.001**	**<0.001**	**0.004**
Any anxiety disorder	19 (18%)	27 (26%)	30 (17%)	1.62, (0.84,3.14)	0.95, (0.50,1.79)	0.2	0.5	>0.9	0.2
Cluster A PD	26 (24%)	26 (25%)	18 (10%)	1.04, (0.56,1.94)	0.35, (0.18,0.68)	**0.001**	>0.9	**0.006**	**0.004**
Cluster B PD	103 (95%)	27 (26%)	4 (2.2%)	0.02, (0.01,0.05)	0.00, (0.00,0.00)	**<0.001**	**<0.001**	**<0.001**	**<0.001**
Cluster C PD	35 (32%)	44 (42%)	41 (23%)	1.50, (0.86,2.63)	0.62, (0.37,1.06)	**0.004**	0.5	0.2	**0.003**

Abbreviations: BGHA, Brown–Goodwin Life History of Aggression; BIS, Barratt Impulsiveness Scale; CI, 95% confidence interval; OR, odds ratio; PD, personality disorder; SA, suicide attempt; SIS, Beck Suicidal Intent Scale.

*Note:* Data in distribution columns are presented as *n* (%) for categorical variables and mean (SD) for continuous variables. Bold type indicates statistical significance at the q < .05 level.

a *q*-values for the global multinomial model were adjusted using the false discovery rate (FDR) correction.

b *q*-values for the pairwise comparisons were adjusted using the Bonferroni correction.

### Suicide pathways within each cluster

**Figure 3.**
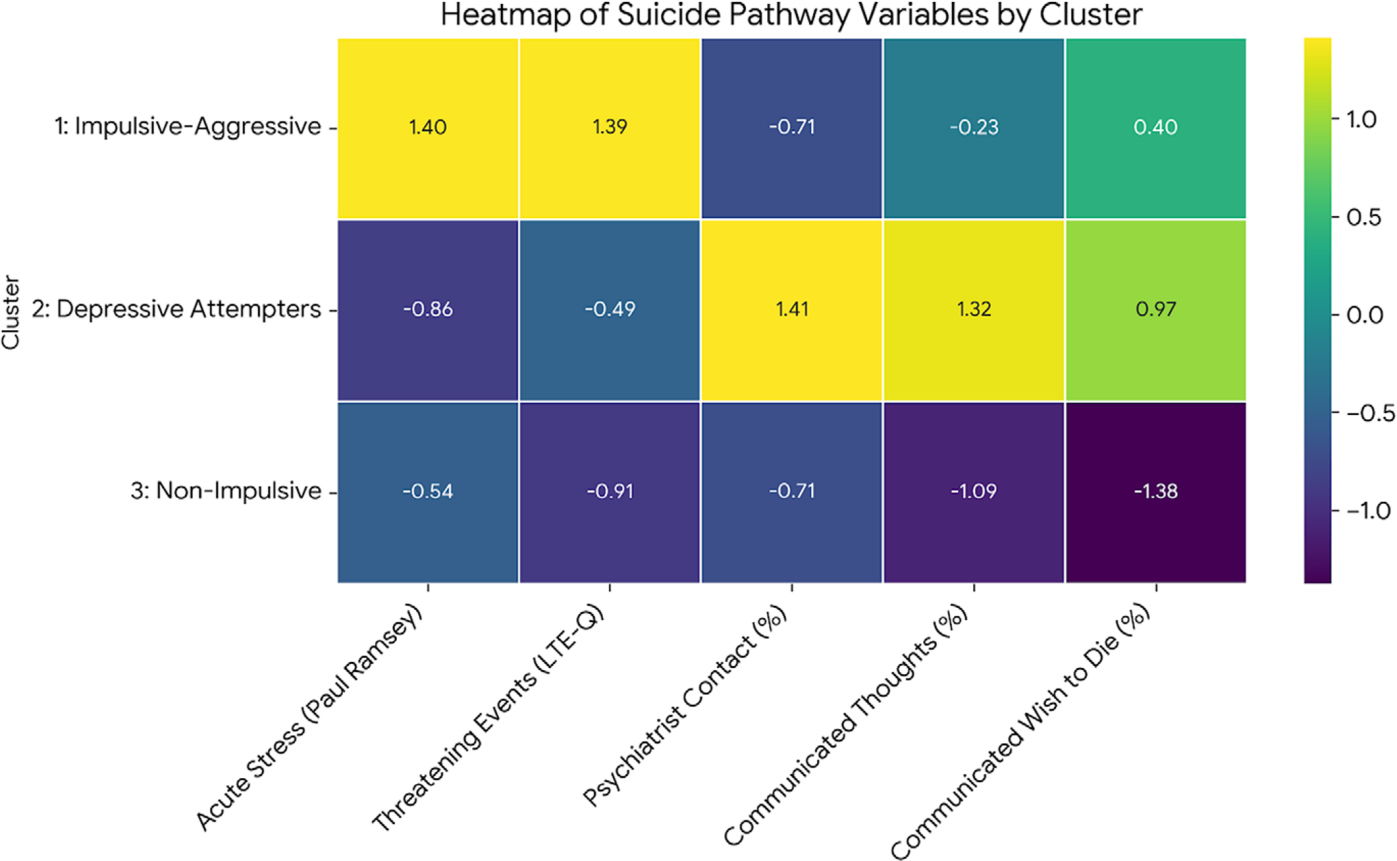
Heatmap of suicide pathway variables by cluster. *Note*: LTE-Q, Life Threatening Events – Questionnaire. This heatmap visualizes the differences in key suicide pathway variables across the three clusters. The values shown are *Z*-scores, which represent for each variable how many standard deviations a cluster’s value is from the mean of all clusters. Positive values indicate a higher-than-average score for that variable, while negative values indicate a lower-than-average score. This allows for a direct comparison of the relative prominence of each pathway characteristic.

Distinct suicide pathways emerged following cluster identification. See Table 2 for full details and Figure 3 for a heatmap visualization of these pathways.

**Table 2.** Suicide pathway between the clusters regarding events in the last year of life

	Distribution			Multinomial model			1 vs. 2	1 vs. 3	2 vs. 3
	Impulsive-aggressive,*N* = 108	Depressive prior attempters,*N* = 105	Non-impulsive-aggressive,*N* = 178	OR (CI)1 vs. 2	OR (CI)1 vs. 3	Global *q*-value^a^	*q*-value^b^	*q*-value^b^	*q*-value^b^
Last year of life									
*Holmes–Rahe Stress Scale*	174 (114)	149 (87)	149 (100)	1.00, (0.99,1.00)	1.00, (0.99,1.00)	0.6	>0.9	0.8	>0.9
*Paul Ramsey Life Experience Scale*	11.6 (5.3)	8.1 (4.6)	8.6 (4.9)	0.87, (0.83,0.93)	0.89, (0.85,0.94)	**<0.001**	**<0.001**	**<0.001**	>0.9
*LTE-Q*	1.79 (1.33)	1.30 (1.02)	1.19 (0.96)	0.69, (0.53,0.88)	0.62, (0.49,0.78)	**<0.001**	**0.013**	**<0.001**	>0.9
Last 3 months of life									
*Had contact with a healthcare professional*	82 (79%)	85 (83%)	130 (74%)	1.34, (0.66,2.71)	0.78, (0.43,1.38)	0.3	>0.9	>0.9	0.3
*Had contact with a psychiatrist 3 months before*	19 (18%)	50 (48%)	32 (18%)	4.26, (2.28,7.96)	1.03, (0.55,1.92)	**<0.001**	**<0.001**	>0.9	**<0.001**
*Communicated thoughts of suicide*	15 (14%)	27 (26%)	13 (7.3%)	2.15, (1.07,4.32)	0.49, (0.22,1.07)	**<0.001**	0.10	0.2	**<0.001**
*Reported suicide intent*	12 (11%)	20 (19%)	10 (5.6%)	1.88, (0.87,4.08)	0.48, (0.20,1.14)	**0.011**	0.3	0.3	**0.002**
Last month of life									
*Communicated a wish to die*	33 (48%)	34 (56%)	29 (23%)	1.37, (0.69,2.74)	0.33, (0.18,0.62)	**<0.001**	>0.9	**0.002**	**<0.001**
*Communicated their desire to die by suicide*	26 (38%)	19 (31%)	17 (14%)	0.75, (0.36,1.55)	0.28, (0.14,0.56)	**0.004**	>0.9	**0.001**	**0.026**
*Commented on any method of suicide*	18 (27%)	14 (23%)	14 (12%)	0.83, (0.37,1.86)	0.36, (0.17,0.79)	0.054	>0.9	**0.032**	0.14
*Gave a reason for taking their life*	21 (31%)	22 (38%)	21 (18%)	1.37, (0.65,2.86)	0.48, (0.24,0.97)	**0.031**	>0.9	0.13	**0.012**
Last 2 weeks of life									
*Kept an interest in their task*						**<0.001**			
All the time	77 (85%)	60 (66%)	135 (93%)	—	—		—	—	—
Nearly always- Rarely	8 (8.8%)	9 (9.9%)	4 (2.8%)	1.44, (0.53,3.97)	0.29, (0.08,0.98)		>0.9	0.14	**0.027**
Never	6 (6.6%)	22 (24%)	6 (4.1%)	4.71, (1.79,12.3)	0.57, (0.18,1.83)		**0.005**	>0.9	**<0.001**
*They were more irritable*						0.2			
Was the same	55 (75%)	51 (74%)	107 (85%)	—	—		—	—	—
With everybody	13 (18%)	16 (23%)	13 (10%)	1.33, (0.58,3.03)	0.51, (0.22,1.18)		>0.9	0.4	0.062
With some people	5 (6.8%)	2 (2.9%)	6 (4.8%)	0.43, (0.08,2.32)	0.62, (0.18,2.11)		>0.9	>0.9	>0.9
*They were angrier*						0.062			
Was the same	64 (77%)	55 (73%)	120 (88%)	—	—		—	—	—
With everybody	15 (18%)	17 (23%)	10 (7.4%)	1.32, (0.60,2.88)	0.36, (0.15,0.84)		>0.9	0.054	**0.007**
With some people	4 (4.8%)	3 (4.0%)	6 (4.4%)	0.87, (0.19,4.07)	0.80, (0.22,2.94)		>0.9	>0.9	>0.9
Last week of life									
Communicated a wish to die	24 (38%)	31 (54%)	23 (20%)	1.99, (0.96,4.11)	0.41, (0.21,0.81)	**<0.001**	0.2	**0.030**	**<0.001**
Communicated their desire to die by suicide	21 (31%)	16 (28%)	15 (13%)	0.85, (0.39,1.85)	0.33, (0.16,0.69)	**0.020**	>0.9	**0.011**	0.055
Commented on any method of suicide	17 (26%)	15 (26%)	13 (11%)	1.01, (0.45,2.25)	0.36, (0.16,0.79)	**0.032**	>0.9	**0.034**	**0.041**
Gave a reason for taking their life	19 (29%)	20 (36%)	17 (15%)	1.35, (0.63,2.89)	0.42, (0.20,0.89)	**0.019**	>0.9	0.071	**0.008**

Abbreviations: CI, 95% confidence interval; LTE-Q, Life-Threatening Events Questionnaire; OR, odds ratio.

*Note:* Data in distribution columns are presented as *n* (%) for categorical variables and mean (SD) for continuous variables. Bold type indicates statistical significance at the q < .05 level.

a *q*-values for the global multinomial model were adjusted using the false discovery rate (FDR) correction.

b *q*-values for the pairwise comparisons were adjusted using the Bonferroni correction.

Cluster 1 (impulsive-aggressive) was notably distinguished by experiencing significantly higher acute life stress in the year preceding death compared to the other clusters.

Cluster 2 (depressive prior attempters) showed markedly greater contact with psychiatric services and more frequent communication of suicidal thoughts and intent to others in the 3 months before death, relative to the other groups.

In contrast, Cluster 3 (non-impulsive/aggressive cases with no prior attempts) communicated significantly less about their distress or suicidal plans in the period leading up to death, including being less likely to express a wish to die or suicidal intentions than the other clusters.

### Sex-segregated analysis

Sex-segregated analyses revealed three male clusters (low, medium, and high impulsivity/aggression). The impulsive-aggressive cluster was highly stable, while the low-impulsivity cluster had more prior attempts, higher personality disorder prevalence, and depression at death.

**Figure 4.**
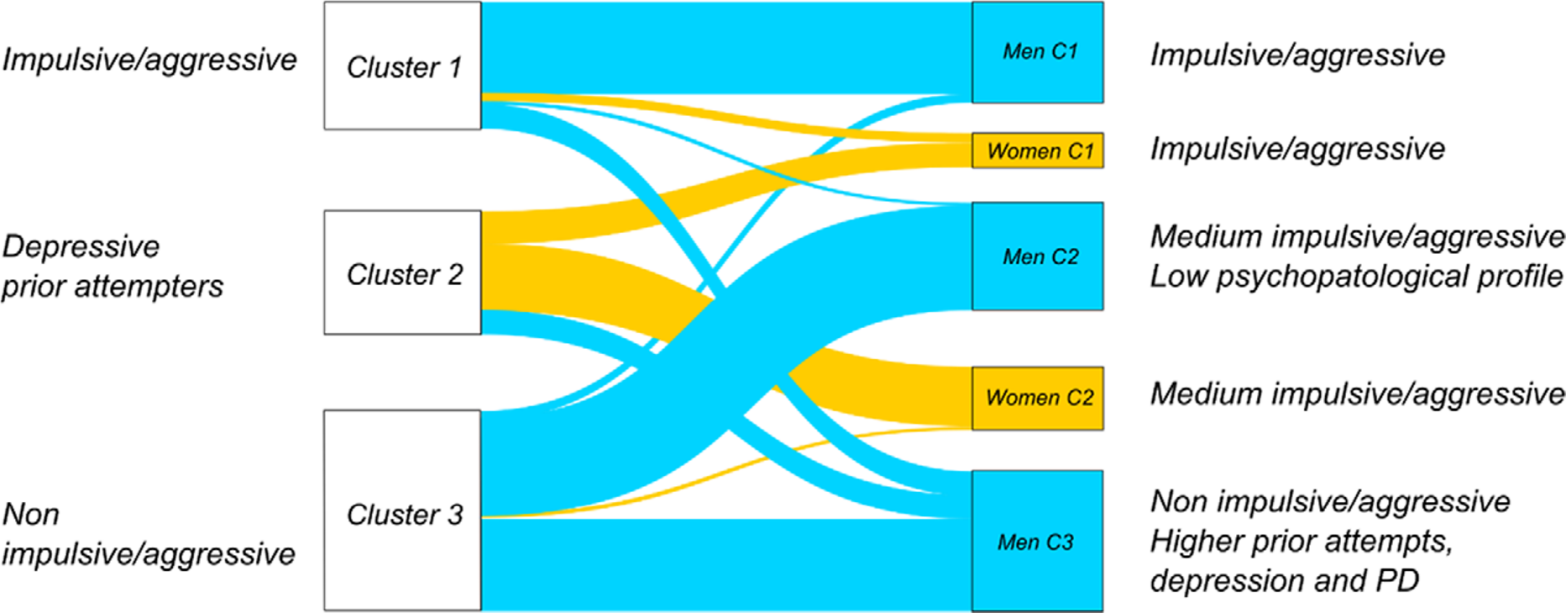
Reorganization of cases in clusters following sex-segregated analysis. *Note*: C, Cluster. This alluvial plot illustrates the flow and reorganization of individuals from the initial three clusters (left nodes) into the new clusters derived from the subsequent sex-segregated analyses (right nodes). The blue bands represent male decedents, and the yellow bands represent female decedents. The plot shows that men were classified into three distinct clusters (Men clusters 1, 2, and 3), while women formed two less-differentiated clusters (Women clusters 1 and 2). The flow demonstrates the stability of certain profiles (e.g., Cluster 1 for men) and the redistribution patterns across the different subgroups.

Women formed two less distinct clusters (Figure 4), but impulsivity/aggression was linked to previous attempts and lower lethal intent (see Supplementary Material).

In male clusters, the non-impulsive-aggressive group had more healthcare contact, suicidal communications, and anger expressions before death, unlike the medium-impulsivity cluster, where these were rare.

Among women, the most impulsive group communicated suicidal intent more often and showed greater anger expressions in the weeks before death than less impulsive/aggressive women (see Supplementary Material).

## Discussion

This study aimed to characterize suicide decedents based on impulsivity and related factors. Our first hypothesis – expecting two groups (high versus low impulsivity) – was partially confirmed. Cluster 1 (impulsive-aggressive) matched expectations, showing high stability across the full sample and male subset. However, among low-impulsivity individuals, we identified two clusters: medium (Cluster 2) and low (Cluster 3) impulsivity, with greater differentiation in men. Both had high suicidal intent, comparable to Cluster 1. Contrary to expectations, depression was more prevalent in the medium-impulsivity cluster, especially in men. In women, clustering was less distinct, although impulsivity/aggression correlated with prior attempts and lower lethal intent.

Our second hypothesis – expecting distinct suicide pathways per cluster – was also partially confirmed. Cluster 1 experienced more negative life events in the year before death, particularly relationship, work, and financial issues. Among women, higher impulsivity was linked to more suicidal communications and anger expressions pre-death. Among men, suicidal communication and behavioural changes were consistent in high- and low-impulsivity clusters, while the medium-impulsivity cluster showed lower rates of both.

### Cluster 1: Impulsive-aggressive substance abusers

A correlation exists between trait impulsivity and lifetime aggression across clusters, with Cluster 1 as the clearest example. This aligns with research linking impulsivity to risky behaviours, including aggression [55, 56]. Impulsivity has been proposed as an endophenotype associated with serotonergic and Hypothalamic-Pituitary-Adrenal axis dysfunction [57, 58], increasing risk for suicidal behaviour, personality disorders, and substance use [12], supporting Cluster 1’s structure.

Additionally, impulsivity/aggression negatively correlate with lethal intent, consistent with prior psychological autopsy studies [28, 29]. However, only extreme impulsivity lowers lethal intent, while medium and low impulsivity clusters show similarly high lethal intent scores.

### Cluster 2: Depressive prior attempters

This cluster is primarily defined by suicide history: 80% had prior attempts, over half had two or more, and 75% experienced depression at death.

Notably, this cluster had a higher proportion of women, although men (one-third of the cluster) had the highest depression and attempt rates. Conversely, women showed greater behavioural changes in the 2 weeks pre-death, including reduced task interest, increased irritability, and anger (see Supplementary Material). Among impulsive women, these changes were more pronounced, with more frequent suicidal thoughts than their less impulsive counterparts.

This pattern in women’s suicides is significant. Anger expression carries higher social costs for women than men [59]. The high proportion of women displaying increased rage pre-suicide suggests a loss of emotional restraint, highlighting emotional dysregulation as a key factor, particularly in high-impulsivity women.

### Cluster 3: Non-impulsive aggressive cases with no prior attempts

The emergence of Cluster 3, the largest profile in our sample, represents a significant challenge for suicide prevention. This profile is not merely defined by an absence of impulsivity or aggression; our data reveal its core characteristics. It is composed almost exclusively of males (99%) for whom the fatal act was their first suicide attempt (80% without prior attempts). Crucially, this profile combines a high, deliberate intent to die (as shown by SIS scores comparable to Cluster 2) with a profound lack of communication regarding this intent. This combination of high internal intentionality and a lack of external warning signs makes this group an empirical manifestation of a “silent” or “invisible” suicide pathway.

This finding is strongly supported by recent large-scale research, which has identified similar “invisible profiles” characterized by a lack of mental health contact and low rates of intent disclosure [60]. In fact, this aligns with foundational work by Logan et al. (2011), who were among the first to empirically identify a subgroup of suicide decedents with few traditional risk factors [61].

To understand the drivers of this silent distress, theoretical frameworks focusing on unbearable psychological pain, or *psychache*, may be more useful than traditional psychopathological models [62]. Furthermore, contemporary ideation-to-action frameworks, such as the Three-Step Theory, can help contextualize this pathway: a combination of pain and hopelessness may escalate suicidal desire, which then progresses to a lethal attempt in individuals with low social connectedness (reflected in their lack of communication) and high capability for suicide [63]. The importance of this “capability” component is underscored by recent findings showing that decedents using high-lethality methods were significantly less likely to have a history of mental health treatment seeking [64]. These theoretical lenses suggest that prevention for this group must expand beyond psychiatric screening to primary care and community settings to identify and alleviate the underlying sources of pain and isolation.

### Gender-specific suicide pathways

Our findings revealed profound gender differences, suggesting distinct suicide pathways for men and women. The clusters showed a stark sex-based separation: Cluster 2 was predominantly female (82%), while Clusters 1 and 3 were almost exclusively male (93 and 99%, respectively). Furthermore, our sex-segregated analyses indicated that impulsivity and aggression served as clearer organizing principles for the male profiles than for the female profiles, suggesting that other factors may be more central to the suicide pathways of women.

The clinical and sociocultural implications of this are significant. The profile of women in Cluster 2, characterized by depression, prior attempts, and pre-death expressions of interpersonal conflict and anger, may reflect gender differences in expressing distress. This may reflect gender-specific pathways to suicide that emerge early in life; for instance, research in adolescents has shown that suicide risk in males is more strongly associated with externalizing behaviours like substance use and impulsivity (mirroring our Cluster 1), while in females, risk is more associated with internalizing symptoms like depression and poor emotional regulation (mirroring our Cluster 2) [65]. Furthermore, women’s internalizing symptoms may manifest as relational turmoil [66], which may explain the increase in anger expressions before death in Cluster 2. Acknowledging these gender-specific patterns of distress is crucial for improving both risk assessment and the therapeutic alliance.

### Implications for further research

This study adds to the growing evidence identifying distinct suicide pathways. Our finding that Cluster 1 was characterized by the highest levels of recent life stressors provides a real-world psychosocial parallel to laboratory findings on biological stress reactivity. For instance, research using the Trier Social Stress Test has demonstrated that high-impulsivity suicide attempters exhibit greater cortisol reactivity [67]. This neurobiological hyper-reactivity may provide a potential mechanism explaining why individuals with an impulsive-aggressive profile are more susceptible to the impact of acute life stressors, which could precipitate a suicidal crisis. Our findings extend this literature, suggesting that impulsivity and aggression vary by sex – more distinct in men and more diffuse in women, indicating possible sex-related biological or psychosocial differences in impulsive suicide pathways.

Consistent with Bernanke et al., who identified stress-responsive and non-stress-responsive suicide attempters [32], our findings align Cluster 1 (impulsive-aggressive) with stress-responsive individuals and Cluster 2 (depressive attempters) with non-stress-responsive individuals. The sex-segregated analysis refines these subtypes: Women in Cluster 2 showed emotional reactivity (irritability and anger) pre-death, while men exhibited depression and prior attempts, aligning with the depressive diathesis model. A history of depression correlates with higher lethal intent and attempt severity [68], consistent with our results.

Cluster 3 lacked a distinct profile, which aligns with its low visibility – it does not stand out in personality, psychopathology, healthcare use, or suicidal communication. However, among men, this cluster split into two: (i) one with low impulsivity but prior depression and attempts, and (ii) another with medium impulsivity but minimal psychopathology. These subgroups highlight suicide risk among men who evade clinical detection.

This hidden group is challenging to identify, as clinical samples underrepresent them. Psychological autopsy is crucial for understanding suicide beyond clinical settings. Given this group’s size and invisibility, further research must examine stressors and life experiences contributing to suicidality, considering sex-specific differences. Community-based strategies, rather than sole reliance on clinical indicators, are essential for detecting individuals at risk.

### Implications for clinical practice

Our findings, by identifying three distinct profiles of suicide decedents, underscore the need for tailored prevention strategies and help explain the limited sensitivity of some universal risk assessment tools [31]. While traditional risk factors like prior attempts may help identify individuals similar to Cluster 2, they are likely to be less effective for the “impulsive-aggressive” (Cluster 1) and, most notably, the large “non-impulsive” (Cluster 3) profiles.

The profile of Cluster 1 aligns with literature identifying impulsivity, aggression, and substance use as key risks, particularly in male suicides [22] and those with Cluster B traits [23]. This suggests that prevention for this group may require interventions focused on stress management and dual diagnosis. Furthermore, the externalizing behaviours in this profile may undermine the benefits of traditional social support [69], indicating that perceived support, rather than simple social contact, is a more crucial target for intervention prevention [70].

In contrast, Cluster 2 was predominantly female, and its profile, characterized by hostility and interpersonal conflict before death, mirrors findings in depressed women [36]. A key clinical implication arises from the paradox that while this group had high healthcare engagement, they showed very low rates of receiving specific evidence-based treatments. This suggests that for individuals with this profile, prevention strategies must address significant barriers to effective care, such as stigma or fear of negative consequences [71, 72].

Furthermore, women in our Clusters more likely exhibited interpersonal conflict and anger before death than their male counterparts. These observations suggest that certain signs may indicate elevated suicide risk in a gender-specific manner.

Finally, the large “non-impulsive” Cluster 3 poses a unique prevention challenge due to its lack of clear psychopathology or classic warning signs. The “invisible” nature of this group highlights the necessity of developing community-based detection strategies and underscores the need for research that extends beyond clinical samples to better understand the environmental stressors and social determinants that contribute to suicide in this large population. The existence of these distinct groups generates testable hypotheses for future research on the differential effectiveness of interventions across these different suicide phenotypes. This includes therapies such as cognitive behavioural therapy, which targets the maladaptive cognitions that may be prominent in depressive profiles like our Cluster 2, and dialectical behaviour therapy, which focuses on emotional dysregulation and distress tolerance, core features of the impulsive-aggressive profile seen in our Cluster 1 [73].

### Strengths and limitations

This study’s strengths lie in its methodological approach, particularly cluster analysis, where variables were rationally selected based on a prior systematic review [33] . This enabled the identification of distinct suicide profiles, offering a nuanced understanding of suicide pathways. The three-cluster model underscores suicidal heterogeneity, aiding tailored prevention strategies. Findings align with literature on impulsivity, aggression, and suicidal behaviour, reinforcing result validity and reliability.

However, limitations exist. The retrospective design and reliance on psychological autopsies may introduce several potential biases. Beyond simple recall bias, our data are also subject to informant bias, as next-of-kin may not have been aware of all aspects of the decedent’s life (e.g., private help-seeking behaviours), and hindsight bias, where knowledge of the suicide may have influenced the informant’s retrospective appraisal of the decedent’s state of mind. Furthermore, a key limitation is the absence of a non-suicide decedent comparison group. Consequently, our findings are relative and describe differences *between* profiles of suicide decedents, but they cannot be used to identify factors that discriminate individuals who die by suicide from those who do not.

While psychological autopsy is validated [74, 75], its inability to establish causality limits understanding of suicide mechanisms. Another limitation of our study is the exclusion of detailed data on medical comorbidities and full pharmacological histories from the clustering model. While some healthcare variables were described, they were not used to define the profiles. Future research should incorporate this data to provide a more refined understanding of the identified suicide pathways.

## Conclusions

This study identified three suicide clusters, revealing differences in impulsivity and related factors. While the impulsive-aggressive cluster was expected, two additional medium- and low-impulsivity clusters emerged, both with high suicidal intent. Unexpectedly, the medium-impulsivity cluster was linked to depression and prior attempts.

Sex-segregated analysis showed men in the impulsive-aggressive cluster had substance use disorders, while women displayed interpersonal conflict and anger pre-death. In medium- and low-impulsivity clusters, women showed mood/behavioural changes, while low-impulsivity men had depression and prior attempts.

Findings stress the need for tailored suicide prevention and community-based research, particularly for the largest, least understood group, which lacks clear psychopathology or healthcare engagement. This approach could enhance suicide prevention strategies.

## Supporting information

10.1192/j.eurpsy.2025.10070.sm001 Sanz-Gomez et al. supplementary material Sanz-Gomez et al. supplementary material

## Data Availability

The data that support the findings of this study are not publicly available due to their sensitive nature and ethical restrictions related to participant privacy and confidentiality.

## Supplementary material

The supplementary material for this article can be found at http://doi.org/10.1192/j.eurpsy.2025.10070.
