# Supplementary material for: Delineating impulsivity-based pathways to suicide deaths: A cluster analysis
Source: Eur Psychiatry. 2025 Aug 11;68(1):e114. doi: 10.1192/j.eurpsy.2025.10070 (PMC12438984; doi:10.1192/j.eurpsy.2025.10070)
Supplement: Sanz-Gomez et al. supplementary material [file S0924933825100709sup001.docx]

Supplementary material

Clustering by gender

# Males

| **Characteristic** | **Distribution** | | | **Multinomial model** | | | **1 vs 2** | **1 vs 3** | **2 vs 3** |
| --- | --- | --- | --- | --- | --- | --- | --- | --- | --- |
|  | **1 N = 116** | **2 N = 94** | **3 N = 86** | **OR, [CI]**  **1 vs 2** | **OR, [CI]**  **1 vs 3** | **global q-value***^1^* | **q-value***^2^* | **q-value***^2^* | **q-value***^2^* |
| **Age** | 63 (19) | 46 (21) | 48 (15) | 0.96, [0.94,0.97] | 0.96, [0.95,0.98] | **<0.001** | **<0.001** | **<0.001** | >0.9 |
| **BIS Total** | 39 (21) | 47 (23) | 73 (23) | 1.02, [1.00,1.03] | 1.06, [1.05,1.08] | **<0.001** | **0.033** | **<0.001** | **<0.001** |
| **BGHA score** | 10 (12) | 8 (9) | 28 (21) | 0.98, [0.96,1.01] | 1.07, [1.05,1.09] | **<0.001** | 0.6 | **<0.001** | **<0.001** |
| **Number of SA** |  |  |  |  |  | **<0.001** |  |  |  |
| *Two or more* | 18 (16 %) | 5 (5.3 %) | 34 (40 %) | — | — |  |  |  |  |
| *One* | 25 (22 %) | 12 (13 %) | 18 (21 %) | 1.73, [0.52,5.77] | 0.38, [0.17,0.88] |  | >0.9 | 0.070 | **0.038** |
| *None* | 73 (63 %) | 77 (82 %) | 34 (40 %) | 3.80, [1.34,10.8] | 0.25, [0.12,0.50] |  | **0.036** | **<0.001** | **<0.001** |
| **SIS score** | 29 (6) | 25 (7) | 21 (9) | 0.92, [0.88,0.96] | 0.86, [0.83,0.90] | **<0.001** | **<0.001** | **<0.001** | **<0.001** |
| **Depressive disorder -current** | 97 (84 %) | 19 (20 %) | 53 (62 %) | 0.05, [0.02,0.10] | 0.31, [0.16,0.61] | **<0.001** | **<0.001** | **0.002** | **<0.001** |
| **Any psychotic spectrum disorder** | 26 (22 %) | 18 (19 %) | 12 (14 %) | 0.82, [0.42,1.61] | 0.56, [0.27,1.19] | 0.3 | >0.9 | 0.4 | >0.9 |
| **Substance abuse** | 13 (11 %) | 36 (38 %) | 68 (79 %) | 4.92, [2.42,10.0] | 29.9, [13.8,65.1] | **<0.001** | **<0.001** | **<0.001** | **<0.001** |
| **Any anxiety disorder** | 31 (27 %) | 10 (11 %) | 10 (12 %) | 0.33, [0.15,0.71] | 0.36, [0.17,0.78] | **0.003** | **0.014** | **0.030** | >0.9 |
| **Cluster A PD** | 32 (28 %) | 5 (5.3 %) | 14 (16 %) | 0.15, [0.05,0.40] | 0.51, [0.25,1.03] | **<0.001** | **<0.001** | 0.2 | 0.068 |
| **Cluster B PD** | 22 (19 %) | 0 (0 %) | 81 (94 %) | — | 69.2, [25.1,191] | **<0.001** | **<0.001** | **<0.001** | **<0.001** |
| **Cluster C PD** | 61 (53 %) | 12 (13 %) | 16 (19 %) | 0.13, [0.07,0.27] | 0.21, [0.11,0.40] | **<0.001** | **<0.001** | **<0.001** | 0.8 |

*Note.* Data in distribution columns are presented as n (%) for categorical variables and Mean (SD) for continuous variables. BGHA = Brown-Goodwin Life History of Aggression; BIS = Barratt Impulsiveness Scale; CI = 95% Confidence Interval; OR = Odds Ratio; PD = Personality Disorder; SA = Suicide Attempt; SIS = Beck Suicidal Intent Scale. ^1^ q-values for the global multinomial model were adjusted using the False Discovery Rate (FDR) correction. ^2^q-values for the pairwise comparisons were adjusted using the Bonferroni correction.

# Females

| **Characteristic** | **Distribution** | | **Binomial model** | | |
| --- | --- | --- | --- | --- | --- |
|  | **1 N = 60** | **2 N = 35** | **OR** | **95% CI** | **q-value***^1^* |
| **Age** | 58 (17) | 52 (18) | 0.98 | 0.96, 1.01 | 0.2 |
| **BIS Total** | 44 (25) | 61 (26) | 1.02 | 1.01, 1.04 | **0.030** |
| **BGHA score** | 8 (11) | 20 (18) | 1.06 | 1.03, 1.11 | **0.010** |
| **Number of SA** |  |  |  |  |  |
| *Two or more* | 15 (25 %) | 16 (46 %) | — | — |  |
| *One* | 19 (32 %) | 9 (26 %) | 0.44 | 0.15, 1.26 | 0.2 |
| *None* | 26 (43 %) | 10 (29 %) | 0.36 | 0.13, 0.98 | 0.2 |
| **SIS score** | 28 (6) | 24 (9) | 0.93 | 0.87, 0.98 | **0.048** |
| **Depressive disorder -current** | 46 (77 %) | 22 (63 %) | 0.52 | 0.21, 1.28 | 0.2 |
| **Any psychotic spectrum disorder** | 16 (27 %) | 9 (26 %) | 0.95 | 0.36, 2.43 | >0.9 |
| **Substance abuse** | 6 (10 %) | 8 (23 %) | 2.67 | 0.84, 8.85 | 0.2 |
| **Any anxiety disorder** | 13 (22 %) | 12 (34 %) | 1.89 | 0.74, 4.82 | 0.3 |
| **Cluster A PD** | 12 (20 %) | 7 (20 %) | 1.00 | 0.34, 2.79 | >0.9 |
| **Cluster B PD** | 0 (0 %) | 31 (89 %) | — | — | **<0.001** |
| **Cluster C PD** | 17 (28 %) | 14 (40 %) | 1.69 | 0.70, 4.08 | 0.3 |

*Note.* Data in distribution columns are presented as n (%) for categorical variables and Mean (SD) for continuous variables. BGHA = Brown-Goodwin Life History of Aggression; BIS = Barratt Impulsiveness Scale; CI = 95% Confidence Interval; OR = Odds Ratio; PD = Personality Disorder; SA = Suicide Attempt; SIS = Beck Suicidal Intent Scale. ^1^ q-values for the global multinomial model were adjusted using the False Discovery Rate (FDR) correction.

Suicide pathways

# Males

| **Characteristic** | **Distribution** | | | **Multinomial model** | | | **1 vs 2** | **1 vs 3** | **2 vs 3** |
| --- | --- | --- | --- | --- | --- | --- | --- | --- | --- |
|  | **1 N = 116** | **2 N = 94** | **3 N = 86** | **OR [CI] 1 vs 2** | **OR [CI] 1 vs 3** | **global q-value***^1^* | **p-value^2^** | **p-value^2^** | **p-value^2^** |
| **Last year of life** | | | | | | | | | |
| *Holmes- Rahe Stress Scale* | 159 (110) | 140 (91) | 170 (114) | 1.00, [0.99,1.00] | 1.00, [1.00,1.01] | 0.6 | >0.9 | >0.9 | 0.8 |
| *Paul Ramsey Life Experience Scale* | 8.9 (4.9) | 8.6 (4.9) | 11.6 (5.4) | 0.99, [0.93,1.05] | 1.11, [1.05,1.17] | **<0.001** | >0.9 | **0.001** | **<0.001** |
| *LTE-Q* | 1.24 (0.99) | 1.14 (0.85) | 1.89 (1.44) | 0.90, [0.68,1.19] | 1.62, [1.24,2.10] | **<0.001** | >0.9 | **0.002** | **<0.001** |
| **Last three months of life** | | | | | | | | | |
| *Had contact with a healthcare professional* | 97 (84 %) | 61 (67 %) | 66 (80 %) | 0.38, [0.19,0.73] | 0.72, [0.35,1.50] | **0.032** | **0.012** | >0.9 | 0.2 |
| *Had contact with a psychiatrist 3 months before* | 32 (28 %) | 12 (13 %) | 16 (19 %) | 0.38, [0.19,0.80] | 0.60, [0.30,1.18] | 0.051 | **0.031** | 0.4 | 0.8 |
| *Communicated thoughts of suicide* | 20 (17 %) | 3 (3.2 %) | 14 (16 %) | 0.16, [0.05,0.55] | 0.93, [0.44,1.97] | **0.006** | **0.011** | >0.9 | **0.020** |
| *Reported suicide intent* | 14 (12 %) | 2 (2.1 %) | 11 (13 %) | 0.16, [0.04,0.72] | 1.07, [0.46,2.49] | **0.021** | **0.050** | >0.9 | **0.045** |
| **Last month of life** | | | | | | | | | |
| *Communicated wish to die* | 35 (44 %) | 9 (13 %) | 25 (46 %) | 0.19, [0.08,0.43] | 1.08, [0.54,2.17] | **<0.001** | **<0.001** | >0.9 | **<0.001** |
| *Communicated their desire to die by suicide* | 21 (28 %) | 5 (7.4 %) | 21 (39 %) | 0.21, [0.07,0.59] | 1.67, [0.79,3.50] | **<0.001** | **0.009** | 0.5 | **<0.001** |
| *Commented on any method of suicide* | 21 (28 %) | 4 (6.0 %) | 13 (25 %) | 0.16, [0.05,0.50] | 0.84, [0.38,1.88] | **0.004** | **0.004** | >0.9 | **0.019** |
| *Gave a reason for taking their life* | 24 (32 %) | 6 (9.0 %) | 17 (33 %) | 0.21, [0.08,0.55] | 1.03, [0.48,2.20] | **0.004** | **0.005** | >0.9 | **0.006** |
| **Last two weeks of life** | | | | | | | | | |
| *Kept interest in their task* |  |  |  |  |  | 0.3 |  |  |  |
| All the time | 76 (89 %) | 76 (94 %) | 65 (88 %) | — | — |  |  |  |  |
| Nearly always- Rarely | 2 (2.4 %) | 3 (3.7 %) | 6 (8.1 %) | 1.50, [0.24,9.23] | 3.51, [0.68,18.0] |  | >0.9 | 0.4 | 0.7 |
| Never | 7 (8.2 %) | 2 (2.5 %) | 3 (4.1 %) | 0.29, [0.06,1.42] | 0.50, [0.12,2.02] |  | 0.4 | >0.9 | >0.9 |
| *They were more irritable* |  |  |  |  |  | 0.4 |  |  |  |
| Was the same | 58 (78 %) | 57 (88 %) | 47 (77 %) | — | — |  |  |  |  |
| With everybody | 11 (15 %) | 4 (6.2 %) | 11 (18 %) | 0.37, [0.11,1.23] | 1.23, [0.49,3.10] |  | 0.3 | >0.9 | 0.2 |
| With some people | 5 (6.8 %) | 4 (6.2 %) | 3 (4.9 %) | 0.81, [0.21,3.19] | 0.74, [0.17,3.26] |  | >0.9 | >0.9 | >0.9 |
| *They were angrier* |  |  |  |  |  | 0.071 |  |  |  |
| Was the same | 67 (83 %) | 62 (90 %) | 53 (78 %) | — | — |  |  |  |  |
| With everybody | 11 (14 %) | 2 (2.9 %) | 12 (18 %) | 0.20, [0.04,0.92] | 1.38, [0.56,3.37] |  | 0.12 | >0.9 | **0.040** |
| With some people | 3 (3.7 %) | 5 (7.2 %) | 3 (4.4 %) | 1.80, [0.41,7.85] | 1.26, [0.25,6.52] |  | >0.9 | >0.9 | >0.9 |
| **Last week of life** | | | | | | | | | |
| *Communicated wish to die* | 29 (39 %) | 8 (12 %) | 20 (40 %) | 0.21, [0.09,0.51] | 1.03, [0.50,2.15] | **0.001** | **0.002** | >0.9 | **0.003** |
| *Communicated their desire to die by suicide* | 18 (24 %) | 4 (6.0 %) | 17 (32 %) | 0.20, [0.06,0.63] | 1.50, [0.68,3.27] | **0.002** | **0.018** | >0.9 | **0.002** |
| *Commented on any method of suicide* | 16 (22 %) | 6 (9.0 %) | 14 (27 %) | 0.34, [0.13,0.94] | 1.29, [0.56,2.95] | **0.047** | 0.11 | >0.9 | **0.038** |
| *Gave a reason for taking their life* | 20 (27 %) | 6 (9.4 %) | 15 (31 %) | 0.28, [0.10,0.75] | 1.19, [0.54,2.64] | **0.021** | **0.033** | >0.9 | **0.018** |

*Note.* Data in distribution columns are presented as n (%) for categorical variables and Mean (SD) for continuous variables. CI = 95% Confidence Interval; LTE-Q = Life-Threatening Events Questionnaire; OR = Odds Ratio. ^1^ q-values for the global multinomial model were adjusted using the False Discovery Rate (FDR) correction. ^2^q-values for the pairwise comparisons were adjusted using the Bonferroni correction.

# Females

| **Characteristic** | **Distribution** | | **Binomial model** | | |
| --- | --- | --- | --- | --- | --- |
|  | **1 N = 60** | **2 N = 35** | **OR** | **95% CI** | **q-value***^1^* |
| **Last year of life** |  |  |  |  |  |
| *Holmes- Rahe Stress Scale* | 156 (82) | 154 (120) | 1.00 | 0.99, 1.01 | >0.9 |
| *Paul Ramsey Life Experience Scale* | 8.4 (5.1) | 8.3 (4.3) | 1.00 | 0.91, 1.09 | >0.9 |
| *LTE-Q* | 1.39 (1.10) | 1.26 (0.90) | 0.88 | 0.57, 1.34 | >0.9 |
| **Last three months of life** | | | | | |
| *Had contact with a healthcare professional* | 46 (79 %) | 27 (79 %) | 1.01 | 0.36, 2.99 | >0.9 |
| *Had contact with a psychiatrist 3 months before* | 26 (43 %) | 15 (43 %) | 0.98 | 0.42, 2.27 | >0.9 |
| *Communicated thoughts of suicide* | 9 (15 %) | 9 (26 %) | 1.96 | 0.69, 5.62 | 0.9 |
| *Reported suicide intent* | 6 (10 %) | 9 (26 %) | 3.12 | 1.02, 10.2 | 0.7 |
| **Last month of life** | | | | | |
| *Communicated wish to die* | 14 (50 %) | 13 (54 %) | 1.18 | 0.40, 3.57 | >0.9 |
| *Communicated their desire to die by suicide* | 5 (18 %) | 10 (43 %) | 3.54 | 1.03, 13.5 | 0.7 |
| *Commented on any method of suicide* | 5 (17 %) | 3 (13 %) | 0.75 | 0.14, 3.44 | >0.9 |
| *Gave a reason for taking their life* | 8 (29 %) | 9 (41 %) | 1.73 | 0.53, 5.77 | 0.9 |
| **Last two weeks of life** | | | | | |
| *Kept interest in their task* |  |  |  |  |  |
| All the time | 40 (67 %) | 15 (56 %) | — | — |  |
| Nearly always- Rarely | 7 (12 %) | 3 (11 %) | 1.14 | 0.22, 4.72 | >0.9 |
| Never | 13 (22 %) | 9 (33 %) | 1.85 | 0.64, 5.22 | 0.9 |
| *They were more irritable* |  |  |  |  |  |
| Was the same | 36 (80 %) | 15 (65 %) | — | — |  |
| With everybody | 9 (20 %) | 7 (30 %) | 1.87 | 0.57, 5.97 | 0.9 |
| With some people | 0 (0 %) | 1 (4.3 %) | — | — | — |
| *They were angrier* |  |  |  |  |  |
| Was the same | 37 (79 %) | 20 (69 %) | — | — |  |
| With everybody | 9 (19 %) | 8 (28 %) | 1.64 | 0.54, 4.97 | 0.9 |
| With some people | 1 (2.1 %) | 1 (3.4 %) | 1.85 | 0.07, 48.5 | >0.9 |
| **Last week of life** | | | | | |
| Communicated wish to die | 11 (42 %) | 10 (45 %) | 1.14 | 0.36, 3.60 | >0.9 |
| Communicated their desire to die by suicide | 6 (22 %) | 7 (33 %) | 1.75 | 0.48, 6.53 | 0.9 |
| Commented on any method of suicide | 7 (24 %) | 2 (9.1 %) | 0.31 | 0.04, 1.48 | 0.9 |
| Gave a reason for taking their life | 8 (30 %) | 7 (33 %) | 1.19 | 0.34, 4.09 | >0.9 |

*Note.* Data in distribution columns are presented as n (%) for categorical variables and Mean (SD) for continuous variables. CI = 95% Confidence Interval; LTE-Q = Life-Threatening Events Questionnaire; OR = Odds Ratio. ^1^ q-values for the global multinomial model were adjusted using the False Discovery Rate (FDR) correction.
